# Association between *XPD* (Lys751G1n) Polymorphism and Lung Cancer Risk: A Population-Based Study in Iran

**Published:** 2014-10-04

**Authors:** Majid Motovali-Bashi, Hojatollah Rezaei, Fariba Dehghanian, Halimeh Rezaei

**Affiliations:** Genetics Division, Department of Biology, Faculty of Sciences, University of Isfahan, Isfahan, Iran

**Keywords:** *XPD*, Lung Cancer, Polymorphism, Iranian, RFLP, NER

## Abstract

**Objective:**

People are usually susceptible to carcinogenic aromatic amines, present in cigarrette smoke and polluted environment, which can cause DNA damage. Therefore, maintenance of genomic DNA integrity is a direct result of proper function of DNA repair enzymes. Polymorphic diversity could affect the function of repair enzymes and thus augment the risk of different cancers. Xeroderma pigmentosum group D (*XPD*) gene encodes one of the most prominent repair enzymes and the polymorphisms of this gene are thought to be of importance in lung cancer risk. This gene encodes the helicase, which is a component of transcription factor IIH and an important part of the nucleotide excision repair system. Studies reveal that individuals with *Lys751Gln* polymorphism of *XPD* gene have a low repairing capacity to delete the damages of ultraviolet light among other *XPD* polymorphisms.

**Materials and Methods:**

In this case-control study, first *Lys751Gln* polymorphism was genotyped, then its association with lung cancer risk was analyzed. Genomic DNA was extracted from the whole blood sample of 640 individuals from Iran (352 healthy individuals and 288 patients). Allele frequencies and heterozygosity of *Lys751Gln* polymorphism were determined using polymerase chain reaction-restriction fragment length polymorphism method.

**Results:**

According to statistical analyses, lung cancer risk in individuals with *Lys751Gln* polymorphism (Odd Ratio=1.8, 95% Confidence Interval 0.848-3.819) is approximately twice as high as that of *Lys/Lys* genotype, however *751Gln/Gln* genotype did not relate to lung cancer risk (Odd Ratio=0.7, 95% Confidence Interval 0/307-1/595).

**Conclusion:**

This study suggests that heterozygous polymorphism (*Lys/Gln*) increases the sensitivity of lung cancer risk, while homozygous polymorphism (*Lys/Lys*) probably decreases its risk and C allele frequency shows no remarkable increase in the patients.

## Introduction

Lung cancer is one of the most common cancers in the world, killing more than one million people a year (1). The majority of lung cancer is attributed to carcinomas, i.e., the malignant cancers originating from epithelial cells. Based on clinical and pathological properties lung carcinomas are divided into two groups of small cell lung carcinoma and non-small cell lung carcinoma. One of the serious problems in lung cancer is its prognosis in late stages (metastasis), making its treatment very difficult. Therefore, the analysis of effective factors in this kind of cancer is of great significance (2). Factors such as smoking, radon gas, industrial carcinogens, air pollution, cancer treatment record, diet and genetic factors play major roles in lung cancer (3-5). So far, 120 different genes that are associated as effective genetic factors in lung cancer risk have been introduced. Among these genes, oncogenes, tumor suppressor genes, apoptosis regulating genes, telomerase genes and the DNA repair genes deserve special mention (6, 7). The study of DNA repair capacity in patients with lung cancer shows a general defect in DNA repair system, leading to low repair capacity and increase in lung cancer risk (8, 9). Malfunctioning of the enzymes involved in DNA repair or their low expression level can be considered as one of the probable factors of low DNA repair capacity (10). Among repair systems, nucleotide excision repair (NER) is very important. The system is responsible for repairing bulky adducts and Ultraviolet (UV) light-induced DNA damage. Cis-Syncyclobutane pyrimidine dimers, pyrimidine (6-4) pyrimidone photoproducts (6-4PPs) and multi ring aromatic hydrocarbons (caused by the components in smoke) can be mentioned among NER substrates (11). NER enzymes with polymorphic diversity have different functions, affecting lung cancer risk. One of the most important of these enzymes is Xeroderma Pigmentosum group D (*XPD*), a helicase encoded by *XPD* gene. This protein has 761 amino acids and a molecular weight of 86,900 Da which has ATP dependent 5´to 3´ helicase activity. *XPD* protein is a member of transcription factor IIH (TFIIH), the role of which is to unwind DNA around the damaged region. *XPD* sequence changes (point mutation, deletion, insertion, inversion and duplication) are seen generally in different populations (12, 13). The 751st amino acid which is located in the C-terminal of *XPD* protein, is responsible for the interaction of the protein with helicase activators. In this polymorphism, polar *Gln* amino acid with CAG codon (C allele) is replaced by basic *Lys* amino acid with AAG codon (A allele) (14). Therefore, this polymorphism in *XPD* gene, changes the structure of the C-terminal, which in turn increases lung cancer risk by changing the *XPD* protein function (15).

## Materials and Methods

### Study population

The lung cancer patients recruited to this casecontrol and retrospective study included 288 people who were first diagnosed by standard histopathological procedures at chronic respiratory disease research center in Masih Daneshvari Hospital, Tehran, Iran. To avoid any possible errors in choosing primary lung cancer cases, blood collection was always performed under the supervision of a medical oncology specialist. The control groups consisted of 352 cancer free volunteers, unrelated to patients, gender, age and smoking statusmatched, randomly selected among those referring to clinics for regular health check-ups. Prior to blood sample collection, a previously prepared questionnaire was completed during a brief face to face interview to obtain demographic characteristics. Blood sampling was done based on patient consent and an agreement signed between the University of Isfahan and Masih Daneshvari Hospital.

### DNA Genotyping

Genomic DNA was extracted from peripheral white blood cells using the salting out method published by Miller (16). To analyze *Lys751Gln* polymorphism, a distinct region with 476 bp length of *XPD* gene [GenBank: NG-007067], which included the polymorphic site, was amplified with PCR. Primers sequences for amplification of this region are listed as follow:

FP: 5´- ATCCTGTCCCTACTGGCCATTC -3´
RP: 5´- CCACTAACGTCCAGTGAACTGC -3´

Each 25 μl PCR reaction mixture contained 2 μl of each forward and reverse primers (10 pM), 2.5 μl of ×10 solution buffer, 0.5 μl of four mixed dNTPs (10 mM), 0.75 μl of Mgcl_2_ (50 mM), 0.3 μl of 5 u/μl Taq DNA polymerase (Cinnagene, Co., Iran), 2 μl (100-200 ng/μl) of genomic DNA. The amplification reaction was carried out under the following conditions: initial denaturation at 95˚C for 4 minutess, followed by 33 cycles, melting at 95˚C for 30 seconds, annealing at 62˚C for 30 seconds and primer extension at 72˚C for 40 seconds, followed by a final extension at 72˚C for 10 minutes. Then, the amplified products were separated by electrophoresis on a 1% agarose gel and visualized by ethidium bromide staining.

PCR amplified products were digested to determine genotypes. PstI restriction enzyme was used to distinguish the *Lys751Gln* polymorphism. PCR products (5 μl) were digested with 2.5-5 units of PstI enzyme in a 10 μl reaction mixture suggested by the manufacturer for 5 hours of incubation at 37˚C. The digested fragments were analyzed by electrophoresis under the condition of 2% agarose gel for 80 minutes at 45V.

There is one recognition site for PstI enzyme in A allele. Therefore, A allele was expected to display two DNA bands with sizes of 105 and 371 bp, whereas C allele because of an additional recognition site for the enzyme was expected to display three DNA bands with sizes of 63, 105 and 308 bp. Individuals carrying heterozygote alleles were expected to show the combination of both alleles.

### Using Image J software

To prove complete enzyme digestion, the number and intensity of the formed bands were analyzed before and after enzyme digestion and were compared using Image J software. Accuracy of enzyme digestion can be examined by comparing the sum area under curve before and after digestion.

### Statistical analyses

C and A allele frequencies of *Lys751Gln* polymorphism was calculated using the genotype obtained from restriction fragment length polymorphism polymerase chain reaction (PCRRFLP). In the next step, *XPD* genotype distribution frequency difference was analyzed by χ2 test. Next, Odd Ratio (OR) with 95% confidence interval (CI) as the association index of *XPD* genotype polymorphism with lung cancer was calculated in case and control groups. Analysis of all stratified data (based on age, gender, smoking habit and so on) was done with SPSS software version 16. A P value less than 0.05 was considered statistically significant.

## Results

**Fig 1:**
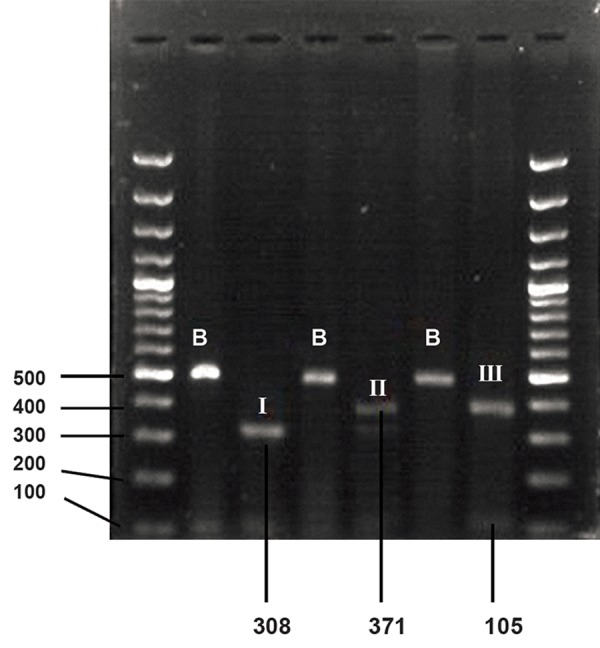
Electrophoresis of PCR-RFLP products with PstI restriction enzyme for analyzing Lys751Gln polymorphism. B; un-digested PCR product, I; recessive homozygous (63, 105, and 308-bp fragments), II; heterozygous (63,105, 308, and 371-bp fragments) and III; dominant homozygous (105 and 371-bp fragments).

Among 288 cases, 13.89% (40 cases) had a diagnosis of small cell carcinoma, 41.66% (120) of adenocarcinoma, 16.67% (48) of squamous cell carcinoma, 11.11% (32) of large cell carcinoma and the remaining 16.67% (48) were of other or unknown histological types. To investigate whether the *XPD* genotype has association with lung cancer, we used individuals who were control and did not carry the C allele (AA homozygote) as a reference group. As mentioned above, in the reference group, the digestion reaction by PstI enzyme results in 63, 105 and 308 bp bands. In contrast, digestion reaction results in two DNA bands with sizes of 105 and 371 bp in CC homozygote individuals and combination of 63, 105, 308 and 371 bands in AC homozygote individuals (Fig 1).

### The association between allelic frequency and genotype of AC polymorphism and lung cancer

In this study, meaningful difference was observed between case and control groups in genotype distribution of AC polymorphism in *Lys751Gln* site (p value=0.047). This shows that probably there exists an association between this polymorphism and lung cancer risk in the study population. However, C allele frequency did not differ significantly between the case and control groups (52.3% in control group and 47.3% in case group, p value=0.525). In other words, C allele frequency in *Lys751Gln* polymorphism was 72.2% in the case and 68.1% in the control group, showing no remarkable increase in the patients in this study. Considering the above allele frequencies, obtained OR was 0.817. It indicates that C allele in *Lys751Gln* polymorphism has no association with lung cancer risk. In general, the potential for lung cancer in AC heterozygous individuals is higher than AA individuals in comparison with CC homozygous ones. The difference can be due to C allele presence beside the A allele (AC) among all affecting factors that alter its activity (Table 1).

### Analysis of RFLP results using Image J Software

**Fig 2:**
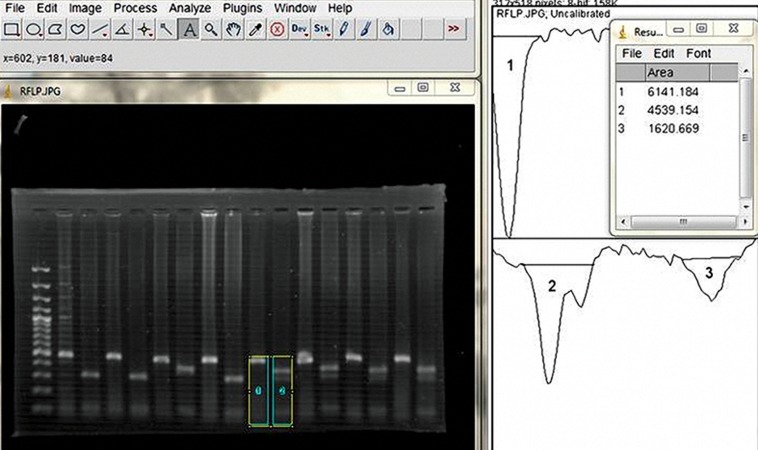
Comparison of the intensity of bands on agarose gel by Image J software. Total area under curve 2 and 3 is equal to the area under the graph 1.

For accurate assessment of enzyme digestion, bands resulting from electrophoresis were evaluated using Image J software. This software has the ability to eliminate the color and brightness in the background gel and compare the intensity of bands. First, gel photo was introduced into the software, and then graphs were drawn based on level of darkness in the diagram window. Measuring the exact area under curve provided three numbers in the result part which showed relatively equal area under the graph. Thus, light emitted from non-restricted band and emitted light resulting from restricted bands were relatively equal and enzyme digestion was properly carried out (4539/154+1620/6 69=6159/823~6141/184) (Fig 2).

**Table 1:** XPD codon 751 polymorphism genotype frequencies and lung cancer risk


Genotype	Controls N (%)	Cases N (%)	P value	OR (CI 95%)

**Total subjects**	352 (100)	288 (100)	-	-
**Lys751Gln**	-	-	-	-
**Lys/Lys**	112 (31.8)	80 (27.7)	-	1.0^a^
**Lys/Gln**	112 (31.8)	144 (50.0)	0.047	1.8 (0.848-3.819)
**Gln/Gln**	128 (36.3)	64 (22.2)	0.525	0.7 (0.307-1/595)
**Gln/Gln + Lys/Gln**	240 (68.1)	208 (72.2)	-	1.213


OR: Odd ratio, CI; Confidence interval and a; Reference value.

## Discussion

The study shows that there is meaningful difference between case and control groups in distribution of AC polymorphism. Although C allele (CC homozygous + AC heterozygous) frequency in the patient group in Iranian population was 4% more than the control group, this difference was not sufficient for an association between the above polymorphism and lung cancer. we suggest that this difference can be due to C allele presence beside A allele (AC) and molecular interaction between them. According to similar results reported by Zhan et al. (17) suggested that the AC polymorphism of *XPD* gene is associated with lung cancer risk and the C allele of *XPD* AC genotype is an increased risk factor for developing lung cancer in a meta-analysis study. Significantly elevated lung cancer risk was associated with C allele, based on Feng et al. (18) study. In contrast, there exists no significant association between having AC heterozygous genotype or CC homozygous genotype with lung cancer patients in different populations. For instance, the studies done in Asia reveal that the average OR for individuals with CC homozygous genotype is 1, whereas it is 0.91 for AC heterozygote genotype individuals. The research in Europe (19, 20) and America (21, 22) showed similar findings. In the above studies, OR is 1.25 for CC homozygous genotype and 1 and 1.04 for AC heterozygous in Europe and America respectively. Also, According to the study by Liang and et al. individuals having CC homozygous genotype show lung cancer about 2.7 times more than individuals with the AA genotype (23).

## Conclusion

The chance to treat lung cancer is so low because of the prognosis of the disease in late stages (metastasis). Therefore, studying this cancer and its related genotype states is of great importance for the early diagnosis of the susceptible individuals. The present study analyzes AC polymorphism frequency in *Lys751Gln* of *XPD* gene in the Iranian population and also its association with lung cancer risk. Our results suggest *Lys/Gln* heterozygous genotype increases lung cancer risk, while *Lys/Lys* homozygous genotype probably decreases its risk and *Gln/Gln* homozygous polymorphism shows no remarkable increase in risk of cancer. These kinds of studies help the experts with timely diagnosis of the cancer and employing effective therapeutic measures particularly in individuals with patients in immediate family members and couples in consanguineous marriages. It is our hope that the association between this polymorphism and lung cancer will be determined more clearly with statistical analyses in diverse populations. In addition, with the completion of profiling the above polymorphism, this technique is suggested as a strong diagnostic biomarker in lung cancer patients.
